# Improved Derivation Efficiency and Pluripotency of Stem Cells from the Refractory Inbred C57BL/6 Mouse Strain by Small Molecules

**DOI:** 10.1371/journal.pone.0106916

**Published:** 2014-09-11

**Authors:** Chih-Jen Lin, Tomokazu Amano, Yong Tang, Xiuchun Tian

**Affiliations:** 1 Department of Animal Science, University of Connecticut, Storrs, Connecticut, United States of America; 2 University of Connecticut Stem Cell Institute, University of Connecticut, Storrs, Connecticut, United States of America; University of Kansas Medical Center, United States of America

## Abstract

The ability of small molecules to maintain self-renewal and to inhibit differentiation of pluripotent stem cells has been well-demonstrated. Two widely used molecules are PD 98059 (PD), an inhibitor of extracellular-signal-regulated kinase 1 (ERK), and SC1 (Pluripotin), which inhibits the RasGAP and ERK pathways. However, no studies have been conducted to compare their effects on the pluripotency and derivation of embryonic stem (ES) cells from inbred mice C57BL/6, an important mouse strain frequently used to model behavior, cognitive functions, immune system, and metabolic disorders in humans and also the first mouse strain chosen to be sequenced for its entire genome. We found significantly increased derivation efficiency of ES cells from in vivo fertilized embryos (fES) of C57BL/6 with the use of PD (71.4% over the control of 35.3%). Because fES and ES from cloned embryos (ntES) are not distinguishable in transcription or translation profiles, we used ntES cells to compare the effect of small molecules on their *in vitro* characteristics, *in vitro* differentiation ability, and the ability to generate full-term ntES-4N pups by tetraploid complementation. NtES cells exhibited typical ES characteristics and up-regulated Sox2 expression in media with either small-molecule. Higher rates of full term ntES-4N pup were generated by the supplementation of PD or SC1. We obtained the highest efficiency of ntES-4N pup generation ever reported from this strain by supplementing ES medium with SC1. Lastly, we compared the pluripotency of fES, ntES and induced pluripotent stem (iPS) cells of C57BL/6 background using the tetraploid complementation assay. A significant increase in implantation sites and the number of full-term pups were obtained when fES, ntES, and iPS cells were cultured with SC1 compared to the control ES medium. In conclusion, supplementing ES cell culture medium with PD and SC1 increases the derivation efficiency and pluripotency, respectively, of stem cells derived from the refractory inbred C57BL/6 strain.

## Introduction

Small molecules have increasingly been applied to ES cell research to improve derivation efficiency and pluripotency maintenance. It has been postulated that the maintenance of ES cells at the ground state is not restricted to the LIF pathway [1], [2]. Rather, this can be achieved by inhibiting pathways that cause ES cell differentiation. Two small molecules have been shown to facilitate ES cell derivation. PD 98059 (PD) is an inhibitor of the extracellular-signal-regulated (ERK) kinase 1 pathway; and SC1 (pluripotin) acts to block the ERK and RasGAP pathways [3], [4]. Recently, both have been used to enhance ES cell derivation in inbred mouse strains such as NOD-SCID and SCID beige that are refractory to ES cell generation [4].

The mouse strain C57BL/6 is the most widely used inbred strain and the first stain chosen for genome sequencing. Although ES cell lines can be obtained using embryos from C57BL/6 mice [5], [6], [7], [8], the low efficiencies of derivation and germ line transmission relatively to ES lines from the 129 strains restricted its wide application in genetic manipulations [9], [10]. Transcription profiling studies showed that ES cells with the C57BL/6 background are more sensitive to culture conditions [11] and have a greater tendency to lose their pluripotency than 129 lines [12]. We hypothesized that adding PD or SC1 to conventional ES culture medium can improve derivation and the pluripotency of ES cells of the C57BL/6 background. First we compared the ES cell derivation efficiencies in PD- or SC1-supplemented ES medium using in vivo fertilized C57BL/6 embryos. Two other types of pluripotent stem cells, ES cells generated from embryos by nuclear transfer (ntES) and induced pluripotent stem (iPS) cells, have been proposed as possessing properties similar to those of ES cells [13], [14], [15], [16]. However, very few studies have been conducted on ntES or iPS cells with the C57BL/6 background [17], [18]. Therefore, in the next experiments we tested the effect of PD or SC1 in the self-renewal and differentiation characteristics of a C57BL/6 ntES cell line. Finally, we compared the pluripotency of all three types of stem cells from C57BL/6: fES, ntES and iPS cultured in the optimal ES medium selected from the first two experiments by subjecting them to the most stringent test for pluripotency, the tetraploid complementation assay. Our results show that PD and SC1 improved the derivation efficiency and pluripotency, respectively, of ES cells from C57BL/6.

## Materials and Methods

### Chemicals

Unless otherwise indicated, chemicals were purchased from Sigma-Aldrich (St. Louis, MO).

### Animals

Experimental mice were purchased from Charles River Laboratories (Wilmington, MA). Animal use and handling procedures were approved by the Institutional Animal Care and Use Committee (IACUC) of the University of Connecticut. CD1 and C57BL/6 strains mice were used as embryo donors by superovulation [19]. Pseudopregnant mice used for recipients were prepared by mating estrous female with vasectomized males.

### ES media and maintenance of pluripotent stem cells (fES, ntES, and iPS cells)

The control ES culture medium was prepared as follows: Knockout-DMEM medium (Invitrogen, Carlsbad, CA) supplemented with 15% Knockout Serum Replacement (KSR; Invitrogen), 100 mM nonessential amino acids, 100 mM 2-mercaptoethanol, 2 mM L-glutamine, 1000 U LIF (Millipore, Billerica, MA) [20]. The ES culture medium was supplemented with small molecules PD 98059 (5.6 µM; Promega, Madison, WI) or SC1 (100 or 300 nM; Pluripotin; Stemgent, Cambridge, MA). These doses were selected based ether on manufacturer’s suggested dose range or from our unpublished preliminary studies. All ES, ntES and iPS cells were maintained in the control ES medium and passaged every 3 days.

### Derivation and maintenance of pluripotent stem cells (fES, ntES and iPS)

For derivation of C57BL/6 ES cell lines from fertilized embryos (fES), we harvested C57BL/6×C57BL/6 *in vivo* fertilized embryos at E1.5 or E3.5 and cultured them *in vitro* until they expanded to hatching blastocysts. Embryos were then placed onto 24-well plates with MEF feeder and cultured in the three types of media (control ES medium, PD98059, and, SC1-300) for 3–4 days for embryonic outgrowth and pickup. After an additional 4–5 days, the colonies were dissociated using 0.05% trypsin-EDTA. The resultant ES-like cells were then passaged into 6-well plates and designated as “passage one”.

The ES cell line from embryos by somatic cell nuclear transfer (ntES) [20] was derived in our lab using male C57BL/6 tail-tip fibroblasts (TTF) as donor cells. The iPS cells used in this study were also derived from male C57BL/6 TTF according to Dr. Yamanaka’s retrovirus protocol [16].

### Flow cytometry analyses for cell cycle distribution and SSEA-1 expression

NtES cells were trypsinized and dissociated into single cell suspensions, then plated onto gelatinized dishes for 30 min to remove feeder cells. For cell cycle analysis, unattached ntES cells were washed twice with DPBS+0.1% BSA, then centrifuged. Cell pellets were resuspended in 75% ethanol solution and stored at −20°C. For staining, cells were pelleted and resuspended in DPBS containing 10 µg/ml of propidium iodide (PI).

For the expression of specific-stage embryonic antigen-1 (SSEA-1), ntES cells were blocked in DPBS with 2% BSA for 15 min at room temperature. Cell lines were divided into two groups and stained with 10 µg/ml mouse IgM monoclonal SSEA-1 antibody (MAB4301, Millipore) or isotype control (IgM negative control, for outer membrane protein 1 of various strains of Neisseria gonorrhoeae, MABC008, Millipore) for 30 min on ice. After being washed in DPBS, cells were stained with the secondary antibody (Alexa Fluor 488 goat anti-mouse IgM, A21042, Invitrogen). Cells were washed and suspended in DPBS for flow cytometry analysis.

Flow cytometry was performed using a FACSCalibur or FACSAriaII flow cytometer (Becton Dickinson, Franklin Lakes, NJ). A total of 10^4^ cells were collected from each batch. Cell debris, dead cells, and doublets were excluded by properly gating on the forward/side scatters. SSEA-1 isotype expression signals were acquired using the FL1-H or FL2-A channel. Cell cycle distributions were computed using the Dean/Jett/Fox module of the FlowJo software (Tree Star, Inc., Ashland, OR).

### Characterization of pluripotent stem cells

Detection of alkaline phosphatase (AP) activity was performed according to the manufacturer’s instruction (alkaline phosphatase substrate kit, Vector Laboratories, Burlingame, CA).

When typical ES cell colonies appeared, immunocytochemistry for pluripotency markers was performed. Briefly, cells were cultivated on glass cover slips with MEF feeder cells for 2–3 days, and fixed in 4% paraformaldehyde (PFA)/1% sucrose solution at 4°C overnight. Fixed samples were then washed in DPBS containing 0.25% Tween-20 (PBST) and treated with 0.5% Triton X-100 for permeabilization. Next, permeabilized samples were washed in PBST. Nonspecific signals were blocked by incubating cells in DPBS plus 2% BSA solution for 1 h at room temperature. ES specific markers, Oct 3/4, Nanog, Sox2 and SSEA-1, were detected with the following specific antibodies: rabbit polyclonal Oct 3/4 (sc-9081, Santa Cruz Biotechnology, Santa Cruz, CA), mouse monoconal Nanog (N3038), rabbit polyclonal Sox2 (ab-5603, Millipore), and SSEA-1 at concentration of 10 µg/ml at 37°C for 2 h. Species and isotype specific secondary antibodies (Alexa Fluor 488,546, Invitrogen) were chosen and incubated at 37°C for 1 h. After being washed in PBST, cell nuclei were counterstained with TO-PRO-3 iodide dye (T3605, Invitrogen) and mounted in ProLong Gold antifade reagent (P36930, Invitrogen). All immunostained images were created using a laser-scanning confocal microscope (Leica TCS SP2, Mannheim, Germany).

In addition to immunostains, RT-PCR and real-time quantitative RT-PCR were also performed as described in our previous report [21]. RT-PCR was used to detect pluripotent markers; real-time RT-PCR was conducted using an ABI Prism 7500 Fast SDS (Applied Biosystems Inc., Foster City, CA) to compare expression levels of pluripotent markers by C57BL/6 ntES cells in different ES media. Cells cultured in control ES medium were used to establish the base level of expression. Relative levels of mRNA of *Oct4*, *Sox2* and *Nanog* were quantified and normalized to the expression level of GAPDH. Two to three biological replicates were conducted. All primer information and amplicon sizes are presented in Table S1.

### Embryoid body (EB) formation and detection of markers for the three germ layers by RT-PCR

NtES cells were trypsinized into single-cell suspensions and plated on gelatin-coated plates for 30 min to remove feeder cells. To create uniform-sized EBs, 3–5×10^5^ unattached ES cells were added to AggreWell plates (Stemcell Technologies, Vancouver, BC, Canada) for 24 h. EBs and cystic EBs formed after 5 days of culture and were cultured for an additional 5 days. Total RNA was extracted and two representative markers from each of the three germ layers were selected for RT-PCR analysis: *Nes* and *Msi1* for ectoderm, *Acta2 (smooth muscle)* and *Bra* for mesoderm, and *Afp* and *Alb* for endoderm.

### Tetraploid complementation assay

Tetraploid (4N) embryos were generated as described in our previous report [20] with slight adjustments. Briefly, 8–12 wk CD1 females were used to produce 4N host embryos. Two-cell embryos were harvested from CD1 mice following superovulation, and electrical pulses (150 V/mm, 50 µsec, 2 pulses) were applied to fuse the two blastomeres [20]. The resultant 4N embryos were cultured to the blastocyst stage in a KSOM+AA medium under 5% CO_2_ for 48 h at 37°C.

ES cells were trypsinized and placed on ice for 30 min before 4N blastocyst microinjection. Fifteen to 20 cells were inserted into the blastocoel of the blastocysts using a piezo injection system (Prime Tech, Japan). The injected blastocysts were cultured for 1–2 h for recovery before being transferred into the uteri of E 2.5 recipient mice. C-section was performed at E 18.5; implantation sites, stillborn fetuses, or live pups were counted; and the weights of the bodies and placentas of 4N pups were recorded.

### Statistical Analysis

qRT-PCR results were analyzed with unpaired t-test. The efficiencies of ES derivation and full-term pup generation were analyzed with Pearson Chi-Square test using the Minitab software. Statistical significance was considered when p<0.05.

## Results

### PD significantly improved the derivation efficiency of fES cells from C57BL/6 mice

**Figure 1:**
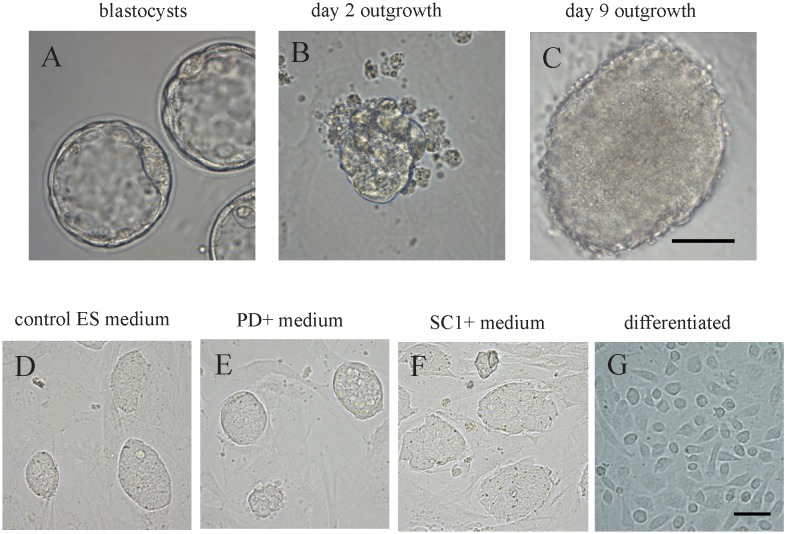
Morphology of newly derived C57BL/6 fES cells cultured in different ES media. A. Expanded blastocysts of C57BL/6 mouse. B, C. Embryonic outgrowth from blastocyst after 2 (B) and 9 days (C) of culture on MEF feeder cells, respectively. D, E, F. ES-like colonies appeared after trypsinization in control ES medium (D), PD- (E) and SC1-supplemented media (F). G. Differentiated colony appeared after trypsinization in control ES medium. Scale bar = 50 µm.

To evaluate the effect of small molecules on ES cell derivation, we used in vivo fertilized embryos because they are of superb quality in general and are therefore more suitable to derive ES cells in a refractory strain. Expanded blastocysts (Fig. 1A) were cultured with feeder cells in three different media: control ES medium, PD- or SC1- supplemented media. Embryonic outgrowths (Fig. 1B) were picked and reseeded onto new feeders. Trypsinization was conducted when outgrowths had proliferated to form clumps of approximately 150–200 µm in diameter (Fig. 1C). fES-like colonies appeared 2–3 days after trypsinization in all media. Interestingly, fES-like colonies derived from the PD- and SC1-supplemented media exhibited morphological differences from typical fES colonies (Fig. 1D) derived in control ES medium. Unlike differentiated colonies, which cannot sustain self-renewal (Fig. 1G), fES cells in the control medium have heterogeneous morphology: some colonies had clear boundaries with multi-cell layers, while others were relative flat. fES colonies derived from PD- and SC1-supplemented media, however, were relatively homogeneous: colonies in PD-supplemented medium all had clear edges (Fig. 1E); while colonies in SC1-supplemented medium were uniformly flat (Fig. 1F).

Supplying either PD or SC1 increased the derivation efficiency of fES cell lines as shown in Table 1. The highest efficiency was obtained from the PD group (71.4%). This was significantly higher than that from the control medium (35.3%). SC1 also improved the rate of fES derivation, although the difference was not significant. Together, these data indicated that PD significantly improved the derivation efficiency of fES cells from the refractory C57BL/6 strain.

**Table 1:** Derivation of ES cell lines in different ES culture media from fertilized embryos of the C57BL/6 strain.

Medium	No. of blastocysts	No. of attachedembryos	No. of outgrowthpicked	No. of welltrypsinization	No. of ES-like colonies(% of blastocysts)
Control−LIF	4	2	1	1	0^a^
Control+LIF	17	15	14	12	6* (35.3%)^a^
PD (5.6 µM)+LIF	14	13	11	10	10 (71.4%)^b^
SC1 (300 nM)+LIF	23	19	16	13	13 (56.5%)^a,b^

*the other 6 outgrowths appeared differentiated after trypsinization.

a,b Values within columns with different superscripts differ, p<0.05.

### Differential effects of small molecules on in vitro characteristics of ntES cells

To characterize the effects of the two molecules on ES cell maintenance, we compared the characteristics of ES cells cultured in three ES media: control ES medium and those supplemented with PD and SC1. In this series of experiments, we used an ES cell line generated from embryos cloned from C57BL/6 fibroblasts [20] because fES and ntES have been shown to be indistinguishable at both the transcriptional and translational levels [22], [23]. Additionally, this cell line has been well-characterized as having a normal karyotype, high expression of pluripotent markers and the capability to generate dopaminergic neurons through target differentiation [24]. Therefore, this ntES line is more suitable for the comparison study.

**Figure 2:**
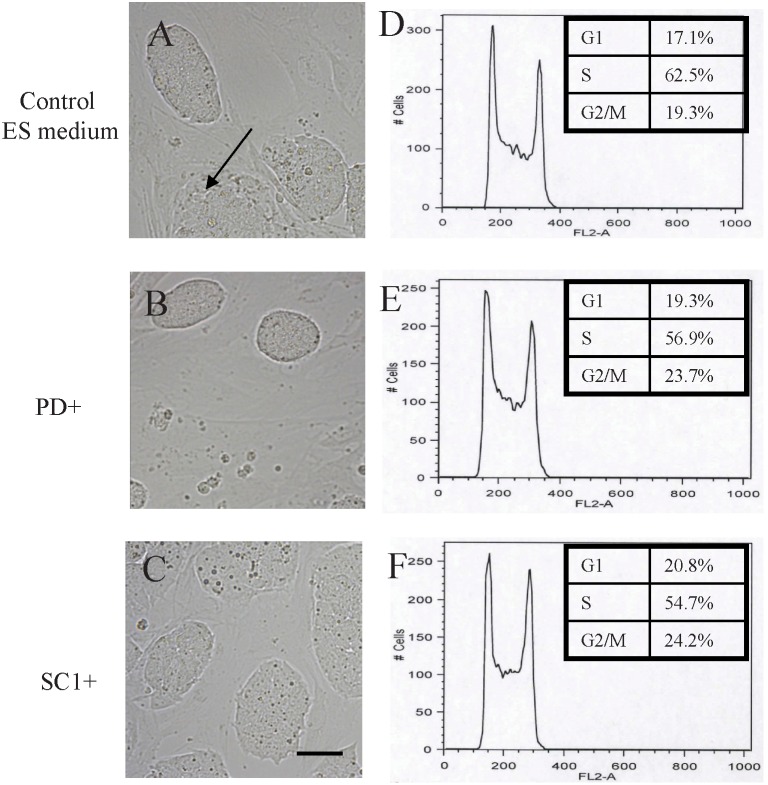
Alteration in morphology and cell cycle distribution of C57BL/6 nuclear transfer ES (ntES) cells cultured in different media. A, B, C. Slight differences of C57BL/6 ntES colonies cultured in control ES medium (A), PD- (B) and SC1-supplemented media (C). Scale bar = 50 µm. Arrow indicates the slight differentiation morphology in control ES medium. D, E, F. Histograms of cell cycle distribution of C57BL/6 ntES cells in the present in control ES medium (D), PD- (E) and SC1-supplemented media (F).

First, we observed that after adapting the ntES cells in different media for 2–3 passages, the cells exhibited morphological changes (Fig. 2A, B, C) similar to those we had noticed during the derivation process (Fig. 1D, E, F), confirming that the small molecule supplements improve ES colony appearance which may be a consequence of pluripotency changes. We wondered if this could be related to the distribution of cells in the cell cycle and conducted flow cytometry analysis. In all three media, a high percentage, 55–63%, of ntES cells was in the S phase. However, for cells cultured in the presence of PD or SC1 the proportions of cells in the S phase were lower, but this difference was not statistically significant and did not change cell growth rate because we were able to passage all cultures at the same schedule of 2–3 passages per week. Therefore the morphological differences of the colonies were not related to cell growth rate or cell cycle distribution changes.

**Figure 3:**
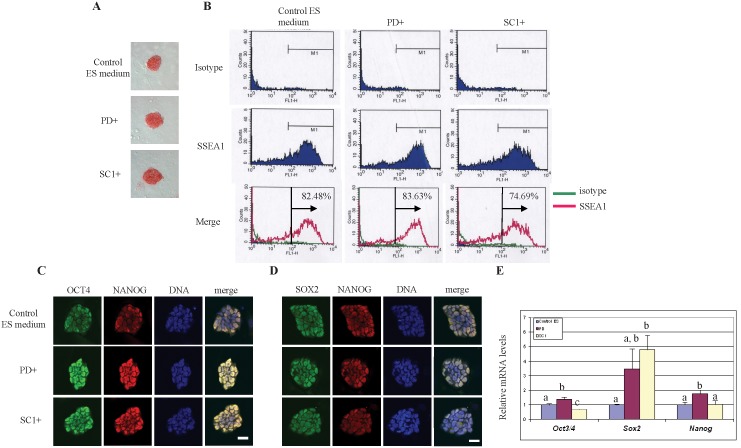
Characterization of C57BL/6 ntES cells cultured in different media. A. Alkaline phosphatase activity of ntES colonies cultured in different ES media. Scale bar = 50 µm. B. SSEA-1 expression profiles of ntES cells cultured in different ES media and characterized by flow cytometry. Pink lines refer to SSEA-1 profiles; green lines refer to negative isotype control. C. Immunocytochemistry detection of ES specific markers Oct4 (green) and Nanog (red) of C57BL/6 ntES cells cultured in different ES media. Scale bar = 25 µm. D. Immunocytochemistry detection of ES specific markers Sox2 (green) and Nanog (red) of C57BL/6 ntES cells cultured in different ES media. E. Effect of small molecules on ES specific marker expression level determined by real-time RT-PCR. ^a,b,c^Values within grouped bar chart with different superscripts differ, *P*<0.05.

We then determined whether the colony morphology changes by small molecules were related to the expression of ES-specific markers by performing a series of ES cell characterizations. NtES cells expressed high activity of alkaline phosphates in all media studied (Fig. 3A). A high proportion (75–84%) of ntES cells showed strong intensity of SSEA-1 as detected by immunochemistry and flow cytometry (Fig. 3B). A somewhat weaker SSEA-1 signal, however, was detected in cells cultured with SC1 (74.7%) than those with PD (83.6%) or in the control ES medium (82.5%).

Typical ES cell pluripotent markers (Oct4, Nanog, and Sox2) were used to assess the in vitro pluripotency of ntES cells under the three culture conditions by both immunocytochemistry and real-time quantitative RT-PCR. NtES cells in all three groups were positively stained for Oct4, Nanog and Sox2 (Fig. 3C, D). Different small molecules, however, had different effects on the relative mRNA levels of these pluripotent genes. *Sox2* was highly up-regulated by both PD and SC1; *Oct4* increased in cells cultured with PD but decreased in those cultured with SC1; *Nanog* was up-regulated in cells cultured with PD and remained unchanged when cultured with SC1 (Fig. 3E). These observations likely reflected the different pathways regulated by PD and SC1.

**Figure 4:**
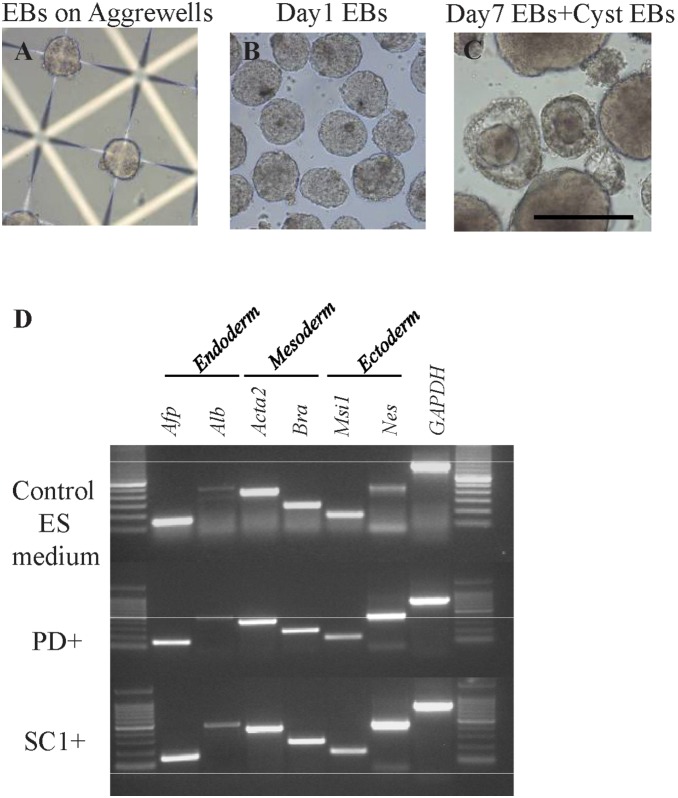
Embryoid body (EB) formation and differentiation of C57BL/6 ntES cells after withdrawal of LIF and SC1 from SC1-supplemented medium. A, B. EBs formation in microwells by C57BL/6 ntES cells 24 h after LIF and SC1 withdraw. C. Morphology of enlarged EBs and cystic EBs after 7 days of culture in suspension. D. RT-PCR analysis of day 10 EBs for markers of the three germ layers.

We also evaluated the *in vitro* differentiation abilities of ntES cells by embryoid body (EB) formation. After removal of LIF, uniform-sized EBs formed in 24 h (Fig. 4A, B) and cystic EBs and enlarged EBs (Fig. 4C) formed after an additional 6 days. After 3 days of differentiation, mRNA of genes representing all three germ-layers was detectable by RT-PCR in all three medium groups (Fig. 4D), demonstrating the pluripotency of these cells.

### SC1 improved pluripotency of ntES cells by tetraploid complementation

The most stringent test for stem cell pluripotency is the tetraploid complementation assay. To investigate whether small molecules improve the *in vivo* developmental potential of ES cells, we used the well characterized ntES line above and compared the three ES media. Upon C-sections at E18.5 we found significantly more implantation sites in the small-molecule supplemented groups than the control group (13.6% of transferred embryos in the control group; 43.5% in PD+ group and 61.8% in SC1+ group; Table 2 and our previous study [25]). Full-term ntES-4N pups were obtained from cells cultured in both supplemented groups, but not from the control group. Significant improvement of full-term pup generation was observed in the SC1-supplemented group. Therefore, the addition of SC1 in ES culture medium enhanced the pluripotency of ntES cells.

**Table 2:** Effects of SC1 on the *in vivo* developmental potential of fES, ntES and iPS cells of the C57BL/6 strain by the tetraploid complementation assay.

Cell type	Medium	Cell passageNo.	No. of embryostransferred	No. ofrecipients	No. of pregnantrecipients	No. of implantation sites(% of transferred embryos)	No. of stillbirths	No. of full-term pups(% of transferred embryos)
fES	Control	P5	86	3	3	5 (5.8)	1	1 (1.2)^a,c^
	SC1 (100 nM)	P3–P4	68	2	2	22 (32.4)^b^	2	3 (4.4)^a,f^
ntES	Control	P6, P7	66	3	1	9 (13.6)^a^	2	0^a,d^
	SC1 (100 nM)	P5 P6	68	3	2	42 (61.8)^b^	0	7 (10.3)^b,g^
iPS	Control	P4	87	3	2	14 (16.1)^a^	7	4^1^ (4.6)^a,e^
	SC1 (100 nM)	P3	89	3	3	40 (44.9)^b^	3	6^2^ (6.7)^a,g^

a,b Comparision of SC1 supplementation within each cell type; data with different superscripts are significantly different (p<0.05).

c,d,e Comparisions among three cell types in control media only; data with different superscripts between cell types are significantly different (p<0.05).

f,g Comparision among three cell types in SC1 supplemented media; data with different superscripts are significantly different (p<0.05).

1 Two of the iPS-4N mice showed umbilical hernia phenotype.

2 One of the iPS-4N mouse showed umbilical hernia phenotype.

### SC1 improved the in vivo developmental potential of fES, ntES and iPS cells

To compare the pluripotency of fES, ntES and iPS and to determine whether small molecule-supplementation enhances their pluripotency, we subjected all three stem cell types to the tetraploid complementation assay. We chose to use SC1 at 100 mM for maintenance culture, because this was the lowest effect dose of SC1 supplementation tested in our study. PD was not used here because it did not significantly improve the in vivo differentiation potential of ntES cells from the above experiment.

**Figure 5:**
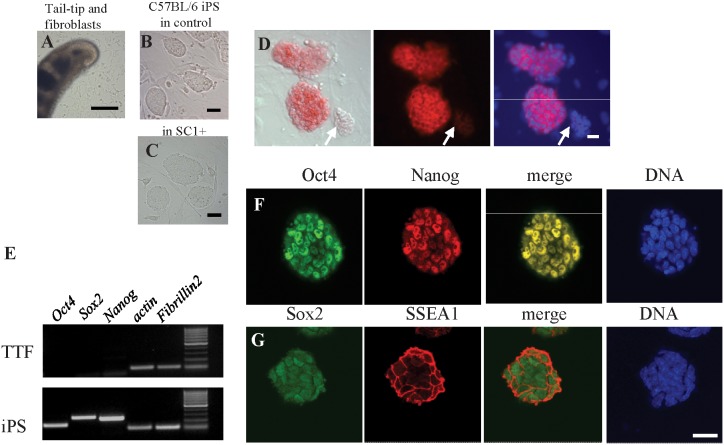
Induction and *in vitro* characterization of iPS cells from C57BL/6 strain. A. Primary culture of fibroblasts from biopsy of tail-tip from C57BL/6 mouse. Scale bar = 200 µm. B, C. Morphology of C57BL/6 iPS colonies cultured in control ES medium (B) and SC1-supplemented medium (C), respectively. Scale bar = 50 µm. D. Alkaline phosphatase activity of C57BL/6 iPS cells. Arrows indicate the weakly stained colonies. Scale bar = 25 µm. E. RT-PCR detection of ES specific and fibroblast markers from C57BL/6 tail-tip fibroblast and iPS cells. F, G. Immunocytochemistry of C57BL/6 iPS cells by Oct4 (green) and Nanog (red; F) and Sox2 (green) and SSEA-1 (red; G) markers, DNA were counterstained by TO-PRO-3 (blue) Scale bar = 25 µm.

FES cells were selected from one of the C57BL/6 lines cultured in SC1-supplemented medium. The iPS cells were generated from C57BL/6 tail-tip fibroblasts that harbor and express the fibroblast marker fibrillin-2 (TTFs, Fig. 5A and 5E; Tang et al., unpublished data) (Fig. 5B) using the conventional 4 factors approach [16]. These cells were alkaline phosphatase positive (Fig. 5D), expressed ES specific markers and are negative for the fibroblast marker fibrillin-2 (Fig. 5E–5G). The slight changes of colony morphology associated with SC1 were also noticed when we switched iPS cells from control ES medium to SC1-containing medium (Fig. 5C).

We found that cells cultured in SC1-supplemented medium formed significantly more implantation sites in the 4N complementation tests (Table 2). In addition, more fES-4N, ntES-4N or iPS-4N full-term pups were generated from cells cultured in SC1-containing medium, although significant improvement was found only in the ntES cell group.

**Figure 6:**
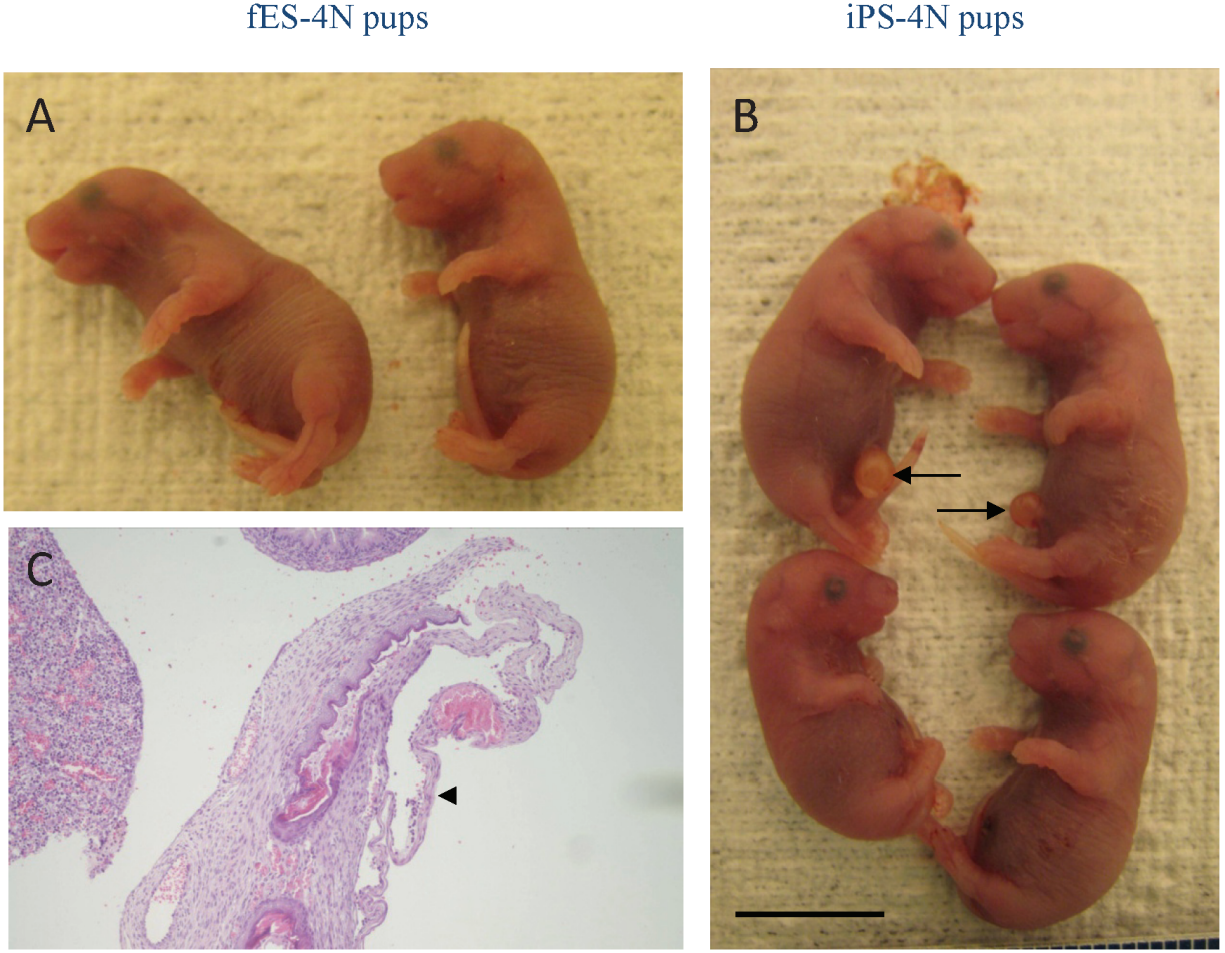
Phenotypes of fES-4N and iPS-4N full-term pups after C-section at E18.5. A. Normal phenotype of fES-4N pups. B. Phenotypes of normal and dilated umbilici found in iPS-4N pups. Arrow indicates region of the dilated umbilicus. Scale bar = 1 cm.

Surprisingly, a high portion (3 out of 10) of the full-term pups derived from iPS cells showed a dilated umbilicus phenotype (Fig. 6B), regardless of the medium used. One iPS-4N mouse was subjected to histological examination, and it was found that the umbilical pouch was composed of skin, a fenestrated ring of fetal mesenchymal tissue that contained dilated fetal blood vessels. There was no intestinal tissue found in the dilated area. This phenotype was not observed in full-term pups derived from fES (Fig. 6A) or ntES cells.

## Discussion

Culture media and culture conditions for ES cells have been constantly modified and improved in the past three decades [26]. Attempts have also been made to improve fES derivation efficiency in mouse strains such as 129, CBA, SCID, and some F1 hybrid strains by supplementing culture medium with small molecules. However, very little has been done in the derivation of fES cells in the inbred C57BL/6 mouse strain, which has served as the model for many fundamental human studies yet is extremely low in ES cell derivation efficiency. Our results demonstrated that supplementing ES culture medium with PD significantly improved the fES derivation rate compared to conventional ES culture medium (Table 1). Although embryonic outgrowths did appear in the control ES medium, they tended to form differentiated colonies after trypsinization (Fig. 1G). Thus, LIF alone is insufficient in the derivation of ES cells from the inbred C57BL/6 and PD is necessary to synergistically maintain pluripotency in C57BL/6 inbred stain as has been reported for other refractory strains [3], [4].

Maintenance of the self-renewal property of ES cells has been proposed to involve not only the LIF/Jak/Stat3 pathway, but also other signaling molecules [1], [2]. This is especially important when the LIF/Jak/Stat3 pathway alone is insufficient to maintain pluripotency as is the case for refractory mouse strains. To determine whether the C57BL/6 mouse stain needs additional pathways for ES pluripotency, we compared the *in vitro* characteristics and *in vivo* developmental potential of ES cells by providing PD or SC1 which activates the ERK and RasGAP as well as ERK Pathways, respectively. These small molecules not only changed the morphology of ES cells but also remarkably up-regulated *Sox2* in both supplemented groups demonstrating their effect in alteration of pluripotency through gene expressions.

The spontaneous in vitro differentiation capability of C57BL/6 ntES cells in regards to EBs formation, however, was not affected by the presence of small molecules. ntES cells cultured in all three media were capable of forming EBs and cystic EBs as well as expressing markers of the multiple germ layers after the withdrawal of LIF and small molecules. Small molecules, however, dramatically improved the pluripotency of ntES cells as determined by the tetraploid complementation assay: cells adapted in either the PD- or SC1-supplemented medium resulted in significantly more implantation sites and full-term ntES-4N pups from both supplemented groups (Table 2). The result from the control medium group was consistent with other reports which have shown that it is extremely difficult to retrieve full-term ntES or ntES-4N pups from the inbred C57BL/6 origin cell lines [27], [28]. To date, only one study reported success in obtaining full-term pups from this cell line [29] using conventional ES medium. Due to the low developmental potential of ES-4N mice and germ-line transmission ability in the C57BL/6 strain, most gene-targeting studies had to be conducted by using F1 hybrid ES cells for homologous recombination. When genetic modifications are needed for human disease models such as cardiovascular or metabolic studies [30] [31], the genetically modified mice need to bred back to C57BL/6 mice for at least 10 generations in order to generate the congenic strains which are comparable to C57BL/6. This process is not only time and economic consuming also delays the research progression. Our results that small molecules increased the full-term rate of ntES-4N pups will greatly enhance the application of gene knockout in this mouse strain.

Our data also support the hypothesis that modification of the ES culture medium with small molecules rescues the quality of fES, ntES, and iPS cell lines in this important mouse strain. Both PD and SC1 inhibit ERK kinase1 activity and prevent pluripotent cell lines from committing to a differentiation path. SC1 also inhibits the RasGAP pathway [2]. Although SC1 is inhibitive to two pathways, we did not observe any significant effect of SC1 over that of PD. From our fES derivation result, it appears that PD-supplementation is more suitable for fES cell derivation (71.4% for PD; 56.5% for SC1; 35.3% for control medium, respectively; data not shown), while SC1-supplementation is better for ES cell maintenance and *in vivo* developmental potentials. The doses for PD (5.6 µM) and SC1 (100 or 300 nM) were effective in these experiments. The concentration of PD used here is much lower than that from other reports (50 µM) [3], [32]. We found this lower dose to be efficient and it also avoids possible cytotoxicity in the processes of fES derivation and maintenance. For SC1, we evaluated the suitable dosage by derivation of parthenogenesis embryonic stem cell lines (data not shown) and chose the optimal range (100–300 nM).

During normal development, umbilical herniation ceases at 16.5 dpc, when the ventral body wall closes completely. Mice display umbilical hernia or omphalocele phenotypes when the ventral body wall fails to close [33]. Although none of our iPS-4N mice (Fig. 6B) displayed a severe form of omphalocele, it would be necessary to confirm the appropriate closure of the ventral body walls using more iPS-4N pups. Because this is an un-expected outcome and it is out of the scope of this study to identify the exact cause for the abnormal morphology of iPS-4N pups. It may be related to the intrinsic characteristics of ES-4N mice, as has been suggested by previous reports [34], [35], although we didn’t observe the hernia phenotype in the rest of our pluripotent cell lines. Another possible explanation is mutagenesis by retrovirus insertions during the iPS induction process. It is known that up to 40 retroviral integration sites might exist in each iPS cell clone [36], and random integration might cause the disruptions of certain genes, resulting in the omphalocele phenotypes such as ROCK-I [37], ROCK-II, or AP-2 alpha [38] genes. Alternatively, this observation could also be caused by the fact that iPS cells retain some epigenetic memories, as has been shown previously [39].

In summary, we demonstrated that all three types of pluripotent stem cells of the C57BL/6 background could generate full-term pups with high efficiency when cultured with SC1 (Table 2). To our knowledge, this is the first report in which fES, ntES and iPS from the same mouse stain were compared simultaneously through the tetraploid complementation assay. Our efficiency in generating full-term iPS-4N pups was much higher than those previously reported without the use of small molecules (4.6–6.7% vs. 1–3.5%) [40], [41], suggesting that SC1 should be a necessary supplement for routine culture of stem cell with the C57BL/6 background.

## Supporting Information

Table S1 Primer sequences and amplicon sizes of PCRs in this study.
(DOCX)
