# Supplementary material for: Improved Derivation Efficiency and Pluripotency of Stem Cells from the Refractory Inbred C57BL/6 Mouse Strain by Small Molecules
Source: PLoS One. 2014 Sep 11;9(9):e106916. doi: 10.1371/journal.pone.0106916 (PMC4161378; doi:10.1371/journal.pone.0106916)
Supplement: Table S1 — Primer sequences and amplicon sizes of PCRs in this study. (DOCX) [file pone.0106916.s001.docx]

Lin et al. **Improved derivation efficiency and pluripotency of stem cells from the refractory inbred C57BL/6 mouse strain by small molecules**

Table S1. Primer sequences and amplicon sizes of PCRs in the study

| Gene | Sequence | Size of amplicon (bp) |
| --- | --- | --- |
| *fibrillin-2* | forward:ACGCAAATCAATTCAGCAGTGT | 108 |
|  | reverse: GGTTGTCCACAGTAAGTTCCGA |  |
| *Sox2* | forward: TAGAGCTAGACTCCGGGCGAT | 300 |
|  | reverse: TTGCCTTAAACAAGACCACGA |  |
| *Nanog* | forward: CTTAGAAGCGTGGGTCTTGG | 265 |
|  | reverse: GACTCCAAGGACAAGCAAGC |  |
| *Oct4* | forward: GAGGATCACCTTGGGGTACA | 189 |
|  | reverse: CTCATTGTTGTCGGCTTCCT |  |
| *Abtinb* | forward: GATGGTGGGAATGGGTCAGA | 167 |
|  | reverse: CGTCCCAGTTGGTAACAATGC |  |
| *GAPDH* | forward: ACCTCAACTACATGGTCTAC | 891 |
|  | reverse: TTGTCATTGAGAGCAATGCC |  |
| *Nes* | forward :AGCAGGAGAAGCAGGGTCTA | 160 |
|  | reverse :TGGGAACTTCTTCCAGGTGT |  |
| *Msi1* | forward :CAGCCAAAGGAGGTGATGTC | 452 |
|  | reverse :GCGCTGATAACTGCTGAC |  |
| *Acta2* | forward :TGTGAAGAGGAAGACAGCACA | 404 |
|  | reverse :ACATACATGGCGGGGACAT |  |
| *Bra* | forward :CCGGTGCTGAAGGTAAATGT | 248 |
|  | reverse :CCTCCATTGAGCTTGTTGGT |  |
| *Afp* | forward :GGCTTTCTAAACACCCATCG | 150 |
|  | reverse :AGTGCGTGACGGAGAAGAAT |  |
| *Alb* | forward :AGGGGACTATCTCCAGGAAA | 464 |
|  | reverse :AGTTGGGGTTGACACCTGAG |  |
